# Pattern Strabismus: Where Does the Brain's Role End and the Muscle's Begin?

**DOI:** 10.1155/2013/301256

**Published:** 2013-06-24

**Authors:** Fatema F. Ghasia, Aasef G. Shaikh

**Affiliations:** ^1^Cole Eye Institute, The Cleveland Clinic Foundation, 9500 Euclid Avenue, Cleveland, OH 44195, USA; ^2^Department of Neurology, Case Western Reserve University School of Medicine, Cleveland, OH 44106, USA

## Abstract

Vertically incomitant pattern strabismus comprises 50% of infantile horizontal strabismus. The oblique muscle dysfunction has been associated with pattern strabismus. High-resolution orbit imaging and contemporary neurophysiology studies in non-human primate models of strabismus have shed light into the mechanisms of pattern strabismus. In this review, we will examine our current understanding of etiologies of pattern strabismus. Speculated pathophysiology includes oblique muscle dysfunction, loss of fusion with altered recti muscle pull, displacements and instability in connective tissue pulleys of the recti muscles, vestibular hypofunction, and abnormal neural connections. Orbital mechanical factors, such as abnormal pulleys, were reported as a cause of pattern strabismus in patients with craniofacial anomalies, connective tissue disorders, and late-onset strabismus. In contrast, abnormal neural connections could be responsible for the development of a pattern in infantile-onset strabismus. Pattern strabismus is likely multifactorial. Understanding the mechanisms of pattern strabismus is pivotal to determine an appropriate surgical treatment strategy for these patients.

## 1. Background

“A” and “V” patterns describe horizontal strabismus that is vertically incomitant and is characterized by a substantial change in the horizontal deviation from the midline position in upgaze as compared to downgaze. Approximately 12–50% of strabismus patients have “A” or “V” patterns [1–4]. “A” and “V” patterns are relatively frequent in patients with infantile-onset strabismus. “A” pattern is present when a horizontal deviation shows a more convergent (less divergent) alignment in upgaze compared with downgaze. The “V” pattern describes a horizontal deviation that is more convergent (less divergent) in downgaze compared with upgaze. The “A” pattern is considered clinically significant when the difference in the measurement between upgaze and downgaze, each approximately 25° from the primary position, is at least 10 prism diopters (Δ). The “V” pattern is considered clinically significant when the difference is at least 15Δ. Other patterns include “Y” where exodeviation is present only in upgaze, lambda (“*λ*”) where exodeviation is present only in downgaze, or “X” where exodeviation is present in downgaze and upgaze compared to the primary position. This review will focus on “A” and “V” pattern strabismus. 

Several theories have been proposed to describe the pathophysiology of “A” and “V” pattern strabismus. Initially it was believed that pattern strabismus is the manifestation of the dysfunction of horizontal and vertical recti muscles [5, 6]. However, since elevation in adduction is frequently associated with the “V” pattern and depression in adduction with the “A” pattern strabismus, it is now believed that the “A” or “V” patterns can be attributed to oblique muscle dysfunction [2]. According to the report of the National Eye Institute sponsored workshop—A Classification of Eye Movement Abnormalities And Strabismus (CEMAS)—superior oblique overaction is referred to as overdepression in adduction while inferior oblique overaction is referred to as overelevation in adduction [7]. Abduction is a tertiary action of obliques. Thus, if the superior oblique is overacting and inferior oblique is underacting it will result in relative divergence in downgaze compared to upgaze, resulting in the “A” pattern. However, “A” and “V” pattern strabismus may be seen without any clinically apparent oblique dysfunction. 

**Figure 1:**
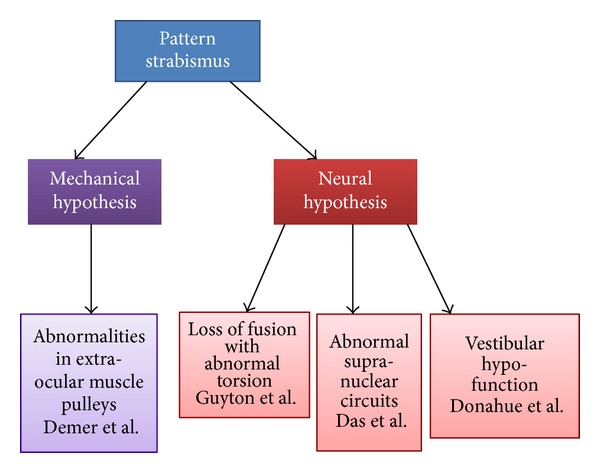
Schematic summary of proposed theories for the pattern strabismus.

The etiology of isolated extraocular muscle (EOM) overaction or underaction is unknown; several hypothetical constructs have been proposed.  Figure 1 depicts the summary of the proposed theories. Broadly, there are two likely sources contributing to pattern strabismus—peripheral and central. The peripheral mechanism suggests mechanical orbital factors induce a gaze position-dependent ocular misalignment resulting in pattern strabismus. In contrast, the central mechanism proposes the role of abnormal neurophysiology as a cause of pattern strabismus. As is often the case, it is possible that pattern strabismus is multifactorial and that it could represent a common phenotype with a combination of central and peripheral etiologies. 

## 2. Mechanical (Peripheral) Etiology of Pattern Strabismus

Increased frequency of the “A” and “V” pattern strabismus has been associated with craniofacial dysmorphisms such as upslanted and downslanted palpebral fissures, plagiocephaly, and hydrocephalus [8–11]. In such cases the strabismus could be related to anomalies in the orientation of superior oblique and inferior oblique muscles, leading to a misdirected muscle force. With the advent of advanced orbital imaging techniques, it was proposed that oblique muscle dysfunction is more a description of the appearance of eye misalignment rather than an actual mechanism of pattern strabismus. High-resolution orbital magnetic resonance imaging (MRI) has demonstrated that, unlike their names, the human recti muscles do not follow straight paths from origins to insertions. In eccentric gaze, the rectus muscle paths are inflected in the anterior orbit. Such inflection is attributed to the EOM pulleys [12]. 

The pulleys have been shown to serve as a functional “origin” of the recti muscles. The pulleys are comprised of annular condensations of the posterior Tenon capsule, composed of collagen and elastin, and stiffened by smooth muscle [13, 14]. The recti have two layers—global and orbital. The global layer of each rectus muscle rotates the globe. The orbital layer inserts on its respective pulley thus moving the pulley posteriorly during contractions. Recti muscle pulleys minimize the sideslip relative to the orbit of posterior muscle paths during globe rotation and determine the effective pulling direction of each rectus muscle. 

Examination of the mechanical properties of the connective tissues defined as pulley bands by van den Bedem in fresh human specimens obtained during exenteration of orbits with no strabismus has shown that the pulleys are unsuited for stabilization of rectus EOM paths [15–17]. However, several other studies using in vivo MRI in humans have shown that orbitally coupled connective tissues are responsible for rectus EOM resistance to sideslips and for sharp EOM path inflections [13, 18–21]. This is in corroboration with finding and persistence of EOM path inflections even after globe enucleation [22] and little alteration of posterior rectus muscle paths after large surgical transpositions [23]. In addition, neural recordings in non-human primates suggest that the orbitally stabilized pulleys can change EOM pulling direction as eye position changes [24, 25]. Thus, there is ample evidence from in vivo imaging, histology, and neurophysiological experiments to support the view that the pulleys can exhibit large shifts and stabilize the posterior EOM rectus paths. The question, then, is whether abnormalities in EOM pulleys can cause pattern strabismus. Displacement of the pulley location, perpendicular to a muscle's plane of action, for example, vertical displacement of horizontal rectus muscle pulley or horizontal displacement of vertical rectus muscle pulley, can cause incomitant strabismus [26, 27]. Heterotopic misplacement of pulleys can produce the clinical appearance of oblique dysfunction, particularly in patients with orbital dysmorphism or connective tissue disorders [28–30].

Although there is a high prevalence of craniofacial anomalies in patients with “A” and “V” pattern strabismus they are not always present [31]. Furthermore, orbital MRI and histologic studies of naturally pattern strabismic non-human primates did not show differences in horizontal recti cross-sectional areas, orbital pulleys, muscle plane paths, innervation densities, or cytoarchitecture compared to the control animals. These animals had neuroanatomical deficits evident as fewer horizontal binocular connections in visual area V1 (striate cortex) [32]. This raises the possibility of abnormal central neurophysiology causing pattern strabismus.

## 3. Neural (Central) Mechanisms of Pattern Strabismus

Abnormal neural connections could cause pattern strabismus by altering the direction of the EOM pull.

### 3.1. Loss of Fusion with Abnormal Torsion

**Figure 2:**
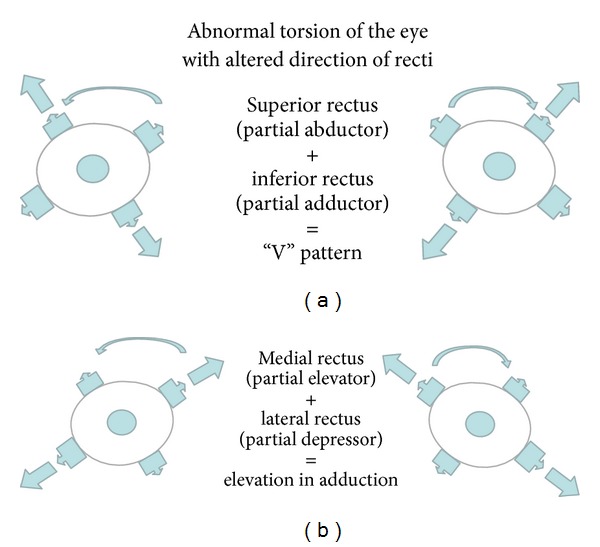
Schematic illustrations of abnormal muscle forces leading to abnormal pull causing “V” pattern strabismus.

It has been proposed that loss of fusion causes pattern strabismus [33]. The authors studied patients with intermittent exotropia overcorrected with horizontal muscle surgery. The comparison group was age-matched controls who maintained fusion postoperatively. About 43% of patients with consecutive esotropia, versus only 5% of controls, developed “A” or “V” pattern at 28 months after surgery [33]. Consequently, the authors suggested that loss of fusion leads to a torsional drift, analogous to exotropic drift, seen in the patients with monocular sensory deprivation. As a result of the torsional drift the pulling direction of the recti muscles are altered mimicking oblique overaction in the nonviewing eye. For example, in “V” pattern strabismus, a tonic torsional shift of the eye with counterclockwise torsion in the right eye and clockwise torsion in the left alters the action of the superior rectus as partial abductor and inferior rectus as partial adductor (Figure 2(a)). Thus pathologically altered force vectors of the vertical recti cause an exoshift in upgaze and an esoshift in downgaze, causing the “V” pattern. By the same mechanism, the medial recti would also function as partial elevators and lateral recti as partial depressors causing vertical deviation on side gaze mimicking inferior oblique overaction (elevation in adduction) (Figure 2(b)). In contrast to this theory a prospective study had reported abnormal torsion on fundus examination years before adduction in elevation (inferior oblique overaction) was seen in infantile esotropia [34]. Hence the role of static torsion in the etiology of pattern strabismus remains a matter of debate. 

### 3.2. Abnormalities in Supranuclear Circuits

Recent studies in non-human primates have suggested the possibility of abnormal supranuclear circuits [35–37]. Newly born macaques reared with alternate monocular occlusion (AMO) during the critical periods of visual development (first 4–6 months of life) develop large horizontal misalignment with “A” or “V” patterns [35]. Behaviorally, these animal models show inappropriate cross-axis movement of the nonviewing eye in the plane orthogonal to the desired eye-movement trajectory. For example, during a desired horizontal task, the nonviewing eye in addition to horizontal movement also has a simultaneous vertical component, causing the covered eye to move in an oblique trajectory [35]. It was proposed that such cross-coupled movements cause ocular misalignment in a gaze position-dependent fashion, as seen in pattern strabismus. 

One putative source of the cross-coupled response could be the altered direction of the recti pull either from the abnormal static torsion or pathologies in the peripheral eye plant. If this were the case, there should be an evidence of some neural correlate of the horizontal cross-coupled response in the activity of the vertical motoneurons and vice versa. However, the animal models of pattern strabismus provide evidence that vertical motoneurons fire in response to vertical eye movements and vertical cross-axis component, and horizontal motoneurons fire in response to horizontal eye movements and horizontal cross-axis component [36, 37]. Thus the animal model refutes the altered recti pull hypothesis. 

 It was subsequently proposed that the cross-coupled responses could be secondary to cross-talk between the horizontal and vertical eye movement areas. Anatomical studies have shown that vertical and torsional eye movement structures project to medial rectus motoneurons [38]. It is possible that such projections could be the source of cross-coupled eye movements. The caveat of this hypothesis is that it is based upon the animal model reared by alternate monocular occlusion. These animals develop strabismus but do not have the classic oculomotor deficits like nasotemporal asymmetry of optokinetic nystagmus (OKN) or latent nystagmus (LN) that is often seen in patients with infantile strabismus. Latent nystagmus has also been studied in animals reared with binocular lid suture without tarsal plate and those reared with prism glasses. There are some important differences between these models. Animals reared with alternate monocular occlusion (AMO) have good visual acuity from each eye but the binocularity is completely disrupted. In contrast, animals reared with binocular lid suture without tarsal plate have thin translucent eyelids which allow diffuse luminance to the retina but prevent spatial vision. Animals reared with prism goggles have binocular image noncorrespondence—thus preventing a normal binocular visual experience but retaining some form of vision from each eye. 

 Mustari and colleagues have shown that nasotemporal optokinetic nystagmus (OKN) asymmetry and latent nystagmus (LN) in monkeys reared with binocular deprivation are due to defects in nucleus of optic tract (NOT). Inactivation of the NOT units using muscimol injections abolishes LN in these animals [39]. Hoffmann and Stone have shown that at birth NOT receives direct input from contralateral retina [40]. Ipsilateral eye inputs from striate and extrastriate visual cortical areas develop with normal binocular visual experience [41, 42]. The NOT units have been shown to have an absence of binocular cells when animals have early onset strabismus [43, 44]. Neuroanatomically, it has been shown that monkeys reared with AMO have an absence of binocular connections between ocular dominance columns of opposite ocularity in the striate cortex whereas other motion processing areas including extrastriate visual cortex-medial temporal area (MT) [45] and subcortical area-NOT [39] have preserved binocular cells [46]. Thus, these animals do not develop latent nystagmus. On the other hand, monkeys with some form vision from each eye such as those reared with binocular lid suture without tarsal plate, the NOT is mainly driven by the contralateral eye with a paucity of cells that respond to both eyes resulting in OKN asymmetry and LN. Monkeys reared with prism goggles develop strabismus, LN, and nasotemporal asymmetry. The severity of strabismus, LN, and OKN asymmetry increases with increasing duration of binocular image noncorrespondence induced by prism goggles [47, 48]. The prism goggles reared animals have been shown to have a loss of binocular connections between ocular dominance columns of opposite ocularity in visual area V1 [49]. This in turn is passed on to extrastriate visual motion processing areas, medial temporal cortex and medial superior temporal cortex (MT and MST). The extrastriate cortex areas project to downstream areas including NOT and other brainstem areas [50]. Hence it would be interesting to investigate whether the cross-coupled responses seen in pattern strabismic monkeys reared with AMO are also seen in animals reared with binocular lid suture without tarsal plate (media opacity model, e.g., congenital cataracts) or those with prism goggles (infantile onset strabismus model) which allows some form of vision from both eyes. 

## 4. Vestibular Hypofunction Theory

In this hypothesis, “A” pattern strabismus was viewed as a special form of “skew deviation” that is seen in patients with brainstem or cerebellar disease [51]. The authors suggested that in “A” pattern strabismus, bilateral brainstem lesions decrease the otolith equivalent of the anterior semicircular canal input. Subsequently, the otolith equivalent of the posterior semicircular canal signals would dominate. Hence there would be activation of the inferior rectus and superior oblique but inhibition of the inferior oblique and superior rectus muscles causing intorsion. Imbalance between the tone of superior and inferior obliques and relative overaction of superior oblique in response to premotor inputs naturally predicts increased ocular tilt responses during head tilt causing otolith stimulation. Absence of such responses in “A” pattern strabismus led to the prediction that the “A” pattern is a special form of skew deviation. While novel, it was based on data from patients who had other neurological deficits including neural tube defects, spinabifida, or hydrocephalus [51]. Such coexisting neurological deficits are not always present in patients with “A” pattern strabismus [52]. 

## 5. Surgical Approach and Outcomes in Pattern Strabismus

The etiology of pattern strabismus, particularly the role of torsion, is the key determinant in the choice of surgery and potential for favorable outcome. For example, surgical treatment for patients with overacting obliques involves weakening of the appropriate obliques, whereas for those without clinically apparent oblique dysfunction it involves horizontal recti transposition to collapse the pattern.

The amount of transposition is determined by the degree of incomitance. It has been shown that horizontal recti transposition collapses the pattern but results in increased objective torsion. Therefore, if ocular torsion drives “A” and “V” pattern strabismus then the surgery that increases the torsion should worsen the pattern strabismus. This is opposite to what is typically seen during horizontal recti transposition [53]. Kushner has reported that rectus muscle transposition surgery for pattern strabismus can cause abnormal ocular torsion, and transposition to address torsion may cause pattern strabismus [54]. Thus, these surgical results suggest that abnormal ocular torsion is less likely to be a cause of pattern strabismus. 

In addition, results of strabismus surgery with similar dosing vary in patients with pattern versus nonpattern exotropia. It has been reported that the postoperative drift in patients with pattern intermittent exotropia was consistently less than in comitant exotropia. The drift was less if the pattern persisted postoperatively and if the exotropia was undercorrected. This suggests differing underlying causes of pattern versus nonpattern exotropia [55, 56].

## 6. Conclusion

Pattern strabismus can have diverse etiologies. Infantile onset strabismus could be due to abnormal neural connections. Late-onset pattern strabismus or pattern strabismus in patients with craniofacial anomalies could be due to orbital pulley instability or abnormal static torsion.
